# Bone marrow mesenchymal stem cell-derived exosomal microRNA-382 promotes osteogenesis in osteoblast via regulation of SLIT2

**DOI:** 10.1186/s13018-023-03667-y

**Published:** 2023-03-10

**Authors:** Hairong Su, Yulan Yang, Wanchun Lv, Xiaoli Li, Binxiu Zhao

**Affiliations:** Maoming People’s Hospital, 101 Weimin Road, Maonan District, Maoming City, 525000 Guandong China

**Keywords:** Osteoporosis, miR-382, SLIT2, Human bone marrow mesenchymal stem cells

## Abstract

**Background:**

Osteoporosis (OP) is a systemic skeletal disorder with increased bone fragility. Human bone marrow mesenchymal stem cells (hBMSCs) have multi-lineage differentiation ability, which may play important roles in osteoporosis. In this study, we aim to investigate the role of hBMSC-derived miR-382 in osteogenic differentiation.

**Methods:**

The miRNA and mRNA expressions in peripheral blood monocytes between persons with high or low bone mineral density (BMD) were compared. Then we collected the hBMSC-secreted sEV and examined the dominant components. The over-expression of the miR-382 in MG63 cell and its progression of osteogenic differentiation were investigated by qRT-PCR, western blot and alizarin red staining. The interaction between miR-382 and SLIT2 was confirmed by dual-luciferase assay. The role of SLIT2 was also confirmed through up-regulation in MG63 cell, and the osteogenic differentiation-associated gene and protein were tested.

**Results:**

According to bioinformatic analysis, a series of differential expressed genes between persons with high or low BMD were compared. After internalization of hBMSC-sEV in MG63 cells, we observed that the ability of osteogenic differentiation was significantly enhanced. Similarly, after up-regulation of miR-382 in MG63 cells, osteogenic differentiation was also promoted. According to the dual-luciferase assay, the targeting function of miR-382 in SLIT2 was demonstrated. Moreover, the benefits of hBMSC-sEV in osteogenesis were abrogated through up-regulation of SLIT2.

**Conclusion:**

Our study provided evidence that miR-382-contained hBMSC-sEV held great promise in osteogenic differentiation in MG63 cells after internalization by targeting SLIT2, which can be served as molecular targets to develop effective therapy.

## Introduction

Osteoporosis (OP) is a systemic skeletal disorder, caused by imbalance of bone metabolism and featured with low bone mass and microarchitectural deterioration in bone tissue, which may result in increased bone fragility [1–3]. Statistics reveal that approximately over 200 million people suffered from OP worldwide and maintain gradual increase every year. In addition, the incidence is significantly higher in female patients than in male patients [4]. Accumulating evidence has illustrated that, as a common metabolic bone disorder, OP progression can be influenced by a variety of factors which may directly or indirectly lead to poor quality of life [5]. At present, therapeutic advance has been made in OP, including hormone replacement therapy, immunotherapy, selective estrogen receptor modulators, bisphosphonates and teriparatide, but limited efficacy and numerous adverse effects still exist. So it is urgent to explore novel treatment strategies for OP.

Human bone marrow mesenchymal stem cells (hBMSCs), identified as multi-potent stromal cells, have multi-lineage differentiation ability and immunosuppressive properties [6–8]. They can be derived from several sources, including the umbilical cord, bone marrow or fat tissue, making them ideal and regeneration as a promising candidate cell type [6, 9]. Accumulating evidence indicates that these features of hBMSCs are associated with extracellular vehicles secretion. Small extracellular vesicles (sEV) are extracellular vesicles generated by fusion with the cellular membrane of multi-vesicular bodies [10–12]. They are between 30 and 150 nm in diameter and contain abundant functional components such as proteins and microRNAs (miRNAs). Currently, hBMSC-derived sEV have been served as an effective strategy for a variety of disease with great characteristics [13]. Nonetheless, the curative effects and mechanisms of the action of hBMSC-sEV on osteogenesis are poorly understood, to the best of our knowledge, particularly in the regulation of osteoblast.

MicroRNA, a predominant component in hBMSC-sEV, is a type of noncoding RNA with a short, single-stranded that target mRNA sequences by binding the 3′-untranslated regions (3′-UTR), which causes degradation or translation blockade of mRNA [14–18]. Previous studies have shown that the miRNA could regulate and affect a variety of cell function, such as proliferation, invasion, inflammation responses and apoptosis [19–21]. A series of miRNAs have been revealed their association with osteogenesis, which participated in the process of bone remodeling, and presented a critical role in the progression of tendon homeostasis and osteoarthritis [22–24]. MiR-20a, miR-27, miR-29b, miR-196a, miR-210 and miR-335-5p have been proved the close relationship with the development of osteoporosis [25–27]. In our present study, we assume the potential role of miR-382 in the regulation of osteogenesis. It has been reported that the miR-382 family is crucial in a variety of cancers, especially in lung cancer, hepatocellular cancer and glioma, presenting significant suppressive function on cell proliferation and metastasis [28–30]. However, the miR-382-associated signaling network has not yet been explored in osteogenesis.

Interestingly, communication between osteoblasts and BMSCs has been identified to take place through small membrane-enclosed vesicular particles, namely exosomes, which is able to fuse with the surrounding cell membranes within circulatory pathways [31, 32]. Exosomes are considered as extracellular vesicles (EVs) and presented great biocompatibility and long-circulating ability [13, 33]. Notably, it has been demonstrated that MSC-derived exosomes exhibited lower immunogenicity and higher levels of regenerative bioactive molecules comparing to those with other cell origins, which emphasized the curative functions of exosomes as the delivery systems of exogenous medications. The current study also reveals that BMSC-derived exosomes may affect the biological properties of human osteoblasts through SATB2 [34]. In order to further explore its potential molecular biological mechanism, we identified by bioinformatics analysis that miR-382 bound to SLIT2.

In our present study, we intended to investigate the effect of hBMSC-sEV on the regulation of osteoblast. Moreover, we investigated the underlying molecular mechanism by using miRNA sequencing of hBMSC-sEV. We hypothesized the potential promoting function of miR-382 and found its targeting mRNA, SLIT2. Our results suggested that hBMSC-sEV can act as a nanotherapeutic agent via miR-382/SLIT2 to promote osteogenic differentiation of osteoblast, which provide a novel therapeutic strategy in the treatment of patients with OP.

## Materials and methods

### Transcriptome data retrieval and preparation

The microarray datasets (GSE62402 and GSE63446) were downloaded from GEO (http://www.ncbi.nlm.nih.gov/geo/) and were used as the training set. These datasets included simultaneously in PBMs from 5 high-hip-BMD subjects and 5 low-hip-BMD subjects. These datasets were produced by an Illumina Humanref-8 V2.0 Expression BeadChip platform. All microarrays from the two datasets were downloaded and normalized and log2 transformed.

### Differentially expressed genes analysis

To identify the differentially expressed genes(DEGs), we used R language (version 3.5.2, edgeR package) analysis among GEO databases. The thresholds were set as log fold change (FC) > 1.5 or 2 along with false discovery rate (FDR) < 0.05.

### Protein protein interaction (PPI) networks

The protein–protein interaction (PPI) network was conducted by the STRING database (https://string-db.org/, version 11.0) and visualized by Cytoscape software (version 3.7.2), and the CytoHubba plug-in in Cytoscape was used to screen the key genes in the PPI network based on the algorithm of maximum neighborhood component (MNC).

### Cell culture and exosome extraction

Human bone marrow mesenchymal stem cell were obtained according to procedures approved by the Ethics Committee at Maoming People's Hospital. hBMSCs were isolated from healthy volunteers and patients with OP. The hBMSCs were cultured under 10% exosome-free FBS for 72 h. The cells, dead cells and debris were removed using several low-speed centrifugations. hBMSCs of passage 5 were used for in our experiments. Cells were cultured in a humidified incubator with 5% CO_2_ at 37 °C and passaged with trypsin/EDTA after reaching the confluence. The human osteosarcoma cell line, MG63 cell, was purchased from ATCC company. MG63 cells were cultured in a humidified incubator with 5% CO_2_ at 37 °C with RPMI 1604 complete medium.

### Isolation of sEV from hBMSC

We followed the MISEV 2018 guidelines to isolate and identify MSC-sEV. Briefly, after MSCs reached 80–90% confluence, the serum-free medium was added for 48 h to avoid contamination of vesicles from serum. The conditioned medium was collected and centrifuged 800 g for 30 min and additional 3000 g for 30 min to remove cells and debris. The supernatant was then subjected to a 0.1-mm-pore polyetherrsulfone membrane filter (Corning) filtration to eliminate cell debris and large vesicles, followed by a 100,000-Mw cutoff membrane concentration (CentriPlus-70, Millipore). The supernatant volume was reduced to less than 5 mL from approximately 250–500 mL. Using the 70Ti Rotor, the supernatant was then ultracentrifuged at 110,000 g for 2 h at 4 °C (Beckman Coulter). The resulting pellets were resuspended with PBS and ultracentrifuged with 100 Ti Rotor for 1 h at 110,000 g at 4 °C (Beckman Coulter). We used PBS buffer as a negative control in the experiments involving hBMSC-sEV.

### Electron microscopy

Transmission electron microscopy (TEM) was performed to detect the size and morphology of EV samples. Isolated EV samples were deposited onto formvar/silicone monoxide-coated 200 mesh copper grids (Electro-microscopy Sciences) for 2–3 min, followed by fixation with 4% formalin and washed twice with water. The samples were contrasted with 2% uranyl acetate (w/v). Then, the grids were visualized with transmission electron microscope (Tecnai G2 Spirit TEM, Zeiss, Oberkochen, Germany) at 120 kV.

### Nanoparticle tracking analysis (NTA)

We measured the EV particle size and concentration using nanoparticle tracking analysis (NTA) with ZetaView PMX 110 (Particle Metrix, Meerbusch, Germany), and corresponding software ZetaView 8.04.02. Isolated EV samples were appropriately diluted using 1X PBS buffer to measure the particle size and concentration. NTA measurement was recorded. The ZetaView system was calibrated using 110 nm polystyrene particles. Temperature was maintained around 23 °C and 30 °C. Size distribution data were analyzed by normalizing the concentration of particles of different diameters with bin widths of 1 nm and then taking the average of each measurement.

### Exosomes internalization assay

MSC-sEV were labeled with a red fluorescent dye (PKH26; Sigma) according to the manufacturer’s instructions. The labeled sEV were then added to MG63 cell and cocultured for 6 h. HUVECs were washed with PBS and fixed in 4% paraformaldehyde for 15 min. Nuclei were stained with DAPI, and the signals were analyzed with a fluorescence microscope.

### Real-time quantitative RT-PCR assay

Total RNA was extracted from each sample with Trizol reagent. Reverse transcription of miRNA was performed using a tailing reverse kit. mRNA was reversed transcribed into first strand cDNA by Prime Script RT kit. The expression of miR-382, SLIT2, ALP, RUNX2 and OCN was detected with SYBR Premix Ex Taq™ (TaKaRa, China) by using Bio-Rad CFX96.

### Western blot analysis

The cells of each group were lysed in lysis buffer. Determine the quality of the harvested protein by used BCA kit. Then, 20 μg of total proteins was separated in SDS-PAGE gels and transferred to PVDF membrane. The membranes were blocked for 1 h at room temperature and incubated overnight at 4 °C with the relevant antibodies: anti-ALP antibody (1:1000, Abcam, USA), anti-OCN antibody (1:1000, Abcam, USA), anti-RUNX2 antibody (1:1000, Abcam, USA), anti-SLIT2 antibody (1:1000, Abcam, USA), anti-CD63 antibody (1:1000, Abcam, USA), anti-TSG101 antibody (1:1000, Abcam, USA), anti-HSP70 antibody (1:1000, Abcam, USA) and anti-GAPDH antibody (1:10,000, Abcam, USA). Membranes were rinsed and incubated for 1 h with secondary antibodies (Abcam, USA). After three times of washing, membranes were exposed with the ECL kit. Bands were analyzed using ImageJ software (version 1.6 NIH) to analyze the relative expression levels of the above markers.

### Cell proliferation assay

We seeded cells (5 × 10^3^) into 96-well culture plates. CCK8 reagents were applied to the culture medium on days 0, 1, 2 and 3 day. We measured the absorbance at 490 nm in each well by a microplate reader after incubation for 1 h at 37 °C (Bio-Rad 680, Hercules, USA), and cell proliferation was represented by each individual well’s mean absorbance minus the blank value of each well.

### Dual-luciferase reporter assay

We first synthesized wild-type and mutant sequences of the SLIT2 3′UTR (untranslated region) containing the miR-382 binding site. These sequences were cloned into the dual-luciferase vector system pmirGLO (Promega, USA). For the dual-luciferase reporter assay, cells were transfected with the WT- or MUT-SLIT2 luciferase reporter plasmid system together with the indicated components. Cells were cultured for another 48 h and collected. A luciferase assay was then carried out using a dual-luciferase reporter assay system (Promega, USA). Experiments were performed according to the manufacturers’ instructions.

### Alizarin red S staining and quantitative analysis

After the in vitro treatment, the culture medium was abandoned and the cells were washed 2–3 times by PBS, fixed in 4% paraformaldehyde for 15 min, washed with dd H_2_O and stained with alizarin red S staining solution (Beyotime) for 30 min. After being rinsed with dd H_2_O, the cells were observed under a microscope (IX50, Olympus, Japan) and photographed. For quantitative analysis, 10% cetyl pyridine chlorophenol (Sigma) was used to dissolve the staining into 10 mm sodium phosphate (Aladdin). The absorbance value was measured at 540 nm using a microplate reader.

### Transfection of mimic and inhibitor

MG63 cell in 6-well plates (5 × 10^5^ cells/well) was transfected with miR-382 mimic (50 nmol/L), or miR-382 inhibitor (100 nmol/L), or their corresponding negative controls. Lipo2000 transfection reagent was simultaneously added into the medium for efficient transfection. After 6 h, we replaced the culture medium in order to remove the transfection reagent. Detection was made 24 h after transfection. miR-382 mimic and miR-382 inhibitor, and their negative controls were purchased from Sangon Biotech.

### Statistical analysis

The data were presented as mean ± SD. Univariate factor analysis of variance (ANOVA) and Student’s *t* test were used for comparison between groups. The data analysis was carried out by SPSS 20.0 statistical software package. When *P* < 0.05, it was considered that the difference was statistically significant.

## Results

### Differentially expressed gene analysis and protein–protein interaction analysis.

**Fig. 1:**
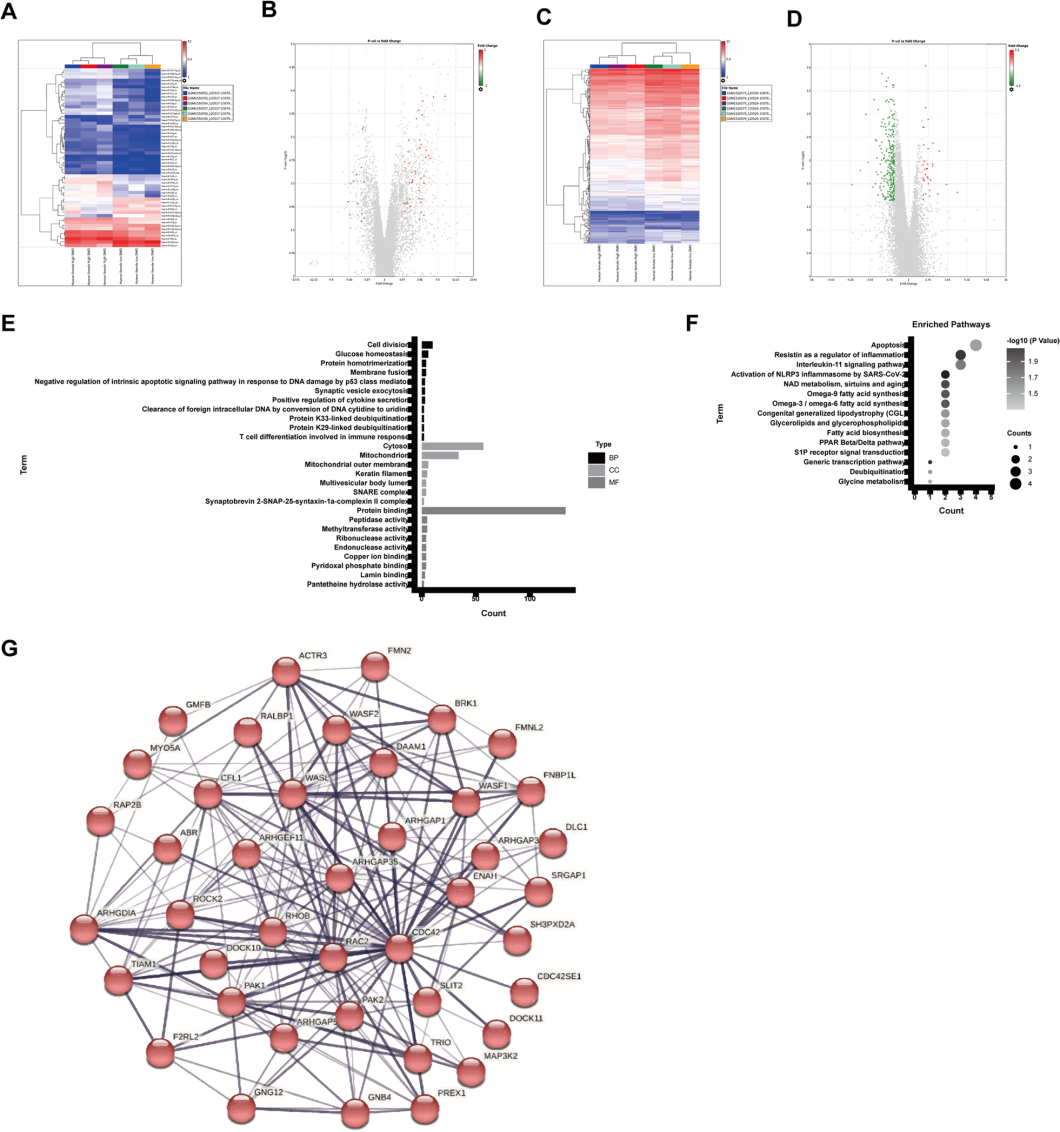
Differentially expressed miRNA gene analysis and protein–protein interaction analysis. **A** Heatmap and **B** volcano plots of DEGs between human samples from OP patients and normal persons. **C** Heatmap and **D** volcano plots of DEGs between human samples from OP patients and normal persons. **E** Top 20 enrichment of GO enrichment analysis and Top 20 enrichment of KEGG enrichment analysis **F**. **G** PPI analysis of DEGs

In order to understand the pathogenesis of osteoporosis (OP), the microarray datasets were downloaded from GEO website, with 5 high bone density (BMD) from healthy subjects and 5 low hip BMD subjects from osteoporosis (OP) patients were included. R language analysis was used to identify the DEGs among these GEO datasets. A total of 54 DEGs (41 up-regulated genes and 13 down-regulated genes) of microRNAs (miRNA) in PBMs were identified from the OP group compared with the normal group, when the log Fold change > 2 or < − 2 along with false discovery rate (FDR) < 0.05, and the DEGs were visualized by the volcano plot and heatmap plot, respectively (Fig. 1A, B). A total of 301 DEGs (33 up-regulated genes and 268 down-regulated genes) of mRNA in PBMs were identified from the OP group compared with the normal group, when the log Fold change > 1.5 or or < − 1.5 along with false discovery rate (FDR) < 0.05, and the DEGs were visualized by the volcano plot and heatmap plot, respectively (Fig. 1A–D). Then GO functional enrichment and KEGG pathway analysis were performed on these DEGs using R software package, and the significant enrichment items of BPs, CCs, MFs were cell division, cytosol and protein binding (Fig. 1E). The KEGG pathway enrichment analysis showed that DEGs were enriched in the apoptosis signaling pathway, resistin as a regulator of inflammation signaling pathway and interleukin-11 signaling pathway (Fig. 1F). Furthermore, we used the STRING database to construct a PPI network for these DEGs with a threshold value of interaction score > 0.4, and the Cytoscape software is used to visualize the PPI network. A node represents a gene, and edges represent relationships between them. Cytoscape software was used to analyze the topology structure of the whole PPI network, and MCC algorithm was used to score the importance of each node in the network (Fig. 1G).

### Characterization and bioinformatic analysis of hBMSC-sEV

**Fig. 2:**
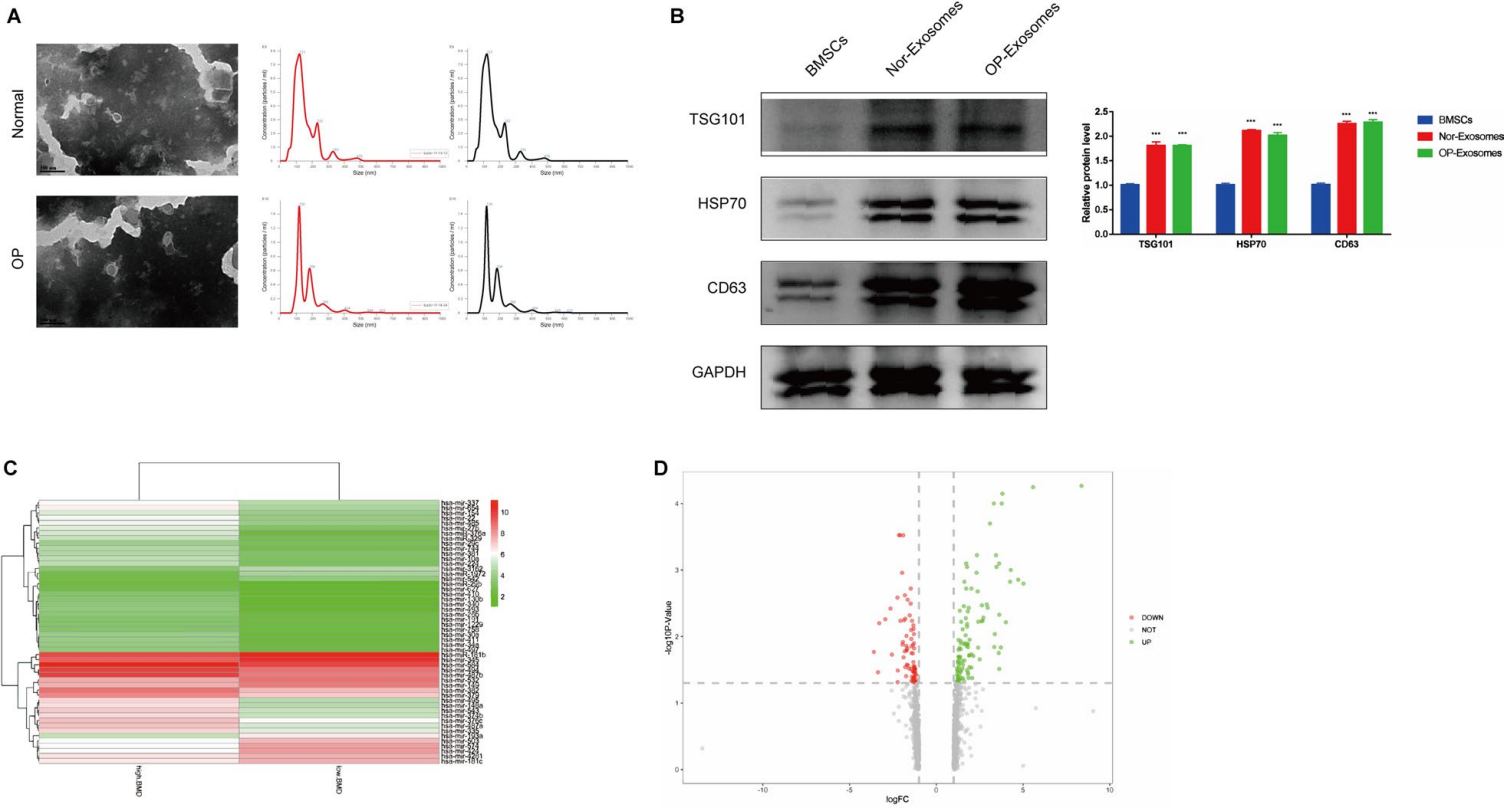
Extraction and identification of exosomes from hBMSCs. **A**. Representative electron microscopy image of hBMSC-sEV from OP patients and normal persons. Bar: 100 nm. The EV particle size and concentration were measured by nanoparticle tracking analysis (NTA). **B** Western blotting analysis of markers (CD63, TSG101, HSP70) in hBMSC-sEV from OP patients and normal persons. **C** Heatmap and **D** volcano plots of DEGs analysis of miRNA between hBMSC-derived small EVs from OP patients and normal persons. Statistical evaluation was performed using one-way ANOVA. Mean ± SEM. *P < 0.05, **P < 0.01, ***P < 0.001

To further explore the biological functions of sEV on osteoporosis, sEV were isolated from supernatant of hBMSCs using ultracentrifugation and characterized by western blot, transmission electron microscopy (TEM) and NTA. Through TEM analysis, hBMSC-sEV exhibited classic cup-shaped or sphere-shaped morphology. In addition, according to NTA, the distribution curve of the particle size of hBMSC-sEV was between 55 and 200 nm (Fig. 2A). Moreover, sEV-associated protein markers CD63 (transmembrane/lipid-bound protein) and TSG101 (cytosolic protein) were enriched in hBMSC-sEV and the negative protein marker HSP70 (an endoplasmic reticulum marker) was not found in hBMSC-sEV compared to hBMSC lysate, which proved the great purity of isolated sEV (Fig. 2B). As a crucial component of exosome cargo, miRNAs have been reported to play critical roles in mediating exosome functions. To identify which miRNA in hBMSC-sEV contributed to osteogenesis, we first performed miRNA sequencing of hBMSC-sEV. The differential expressed miRNAs were analyzed. Overall, we detected 80 mature miRNAs: 26 insignificance gens, 41 up-regulated genes and 13 down-regulated miRNAs (Fig. 2C, D). Then, we chose hsa-miR-382 and hsa-miR-411 for further analysis, as these miRNA are the differentially up-regulated miRNA in exosomes of high-BMD people and exosomes of low-BMD people, which is also the up-regulated miRNA from peripheral blood sequencing analysis of high-BMD people and exosomes of low-BMD people.

### Active internalization of hBMSC-sEV promotes osteogenic differentiation in MG63 cell

**Fig. 3:**
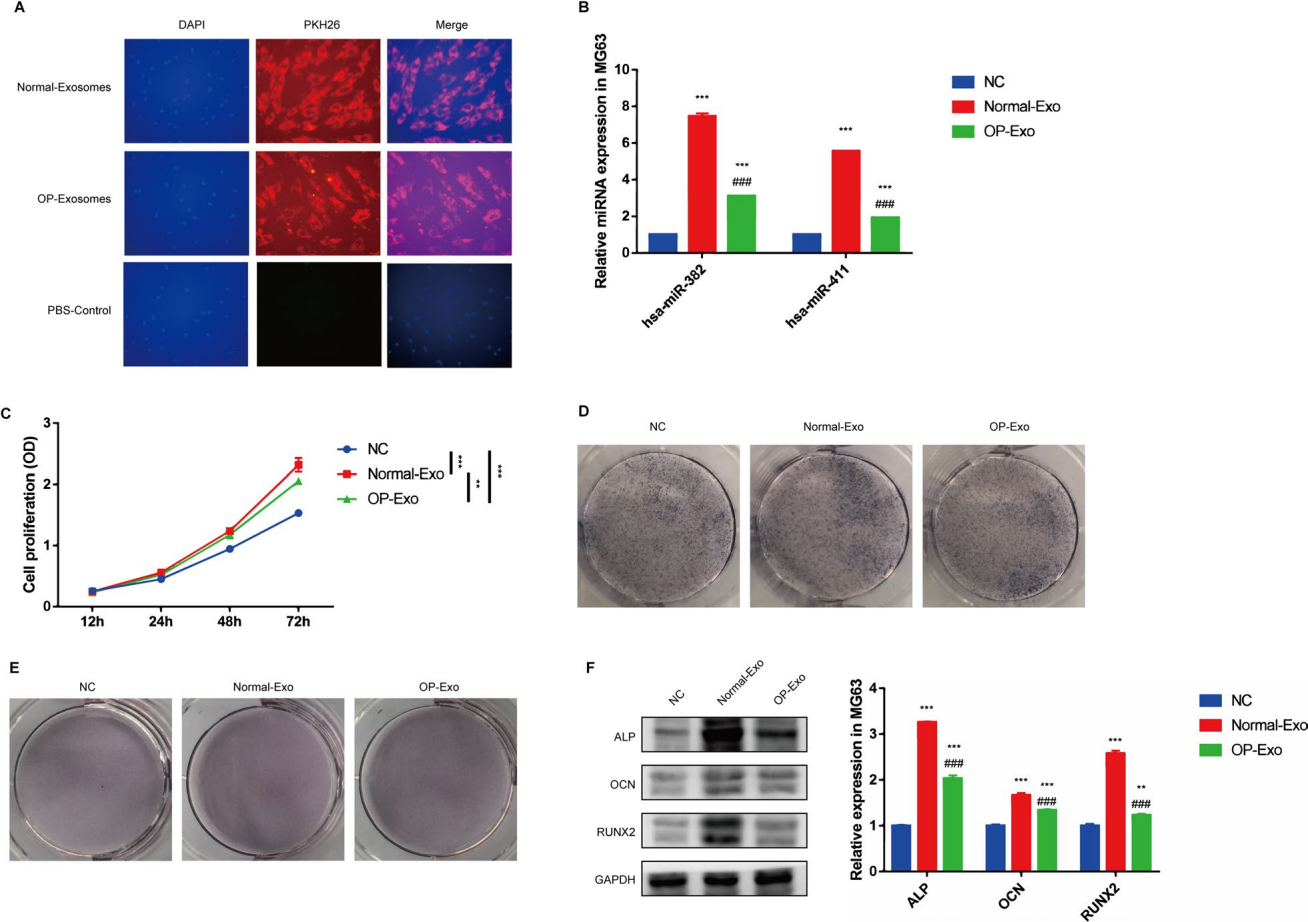
Active internalization of hBMSC-sEV promotes osteogenic differentiation in MG63 cell. **A** Representative microphotographs of immunofluorescence staining of PKH26, **B** mRNA expression of miR-382 and miR-411, **C** cell proliferation, **D** ALP staining, **E** alizarin red S staining and **F** western blotting analysis of osteogenesis-associated protein in MG63 cells treated with PBS or hBMSC-sEV from OP patients and normal persons. Statistical evaluation was performed using two-way ANOVA. Mean ± SEM. *represents *P* < 0.05 compared with NC group. *P < 0.05, ***P < 0.001; # represents *P* < 0.05 compared with HG group. ###*P* < 0.001;

To investigate whether hBMSC-sEV could be internalized by osteoblast,and promote osteogenic differentiation, we used MG63 cell as the cellular model. hBMSC-sEV were labeled with PKH26 and added to the medium of MG63 cells. sEV incorporation can be observed at 2 h after treatment, indicating the internalization of sEV into cells (Fig. 3A). Additionally, the results of qRT-PCR showed that internalization of hBMSC-sEV markedly elevated the expression of miR-382 and miR-411 in MG63 cell (Fig. 3B). Moreover, the internalization of hBMSC-sEV significantly promoted cell proliferation of MG63 cell (Fig. 3C). In terms of osteogenic differentiation, we observed that, according to the ALP staining and alizarin red S staining, the ability of osteogenic differentiation was also remarkably enhanced and the osteogenic differentiation-associated markers (ALP, OCN and RUNX2) were significantly rising compared with untreated group after treatment of hBMSC-sEV (Fig. 3D–F). According to the above results, we hypothesized that active internalization of hBMSC-sEV in osteoblasts may potentially promote the cell proliferation and osteogenic differentiation.

### Up-regulation of miR-382 promotes osteogenesis in MG63 cell

**Fig. 4:**
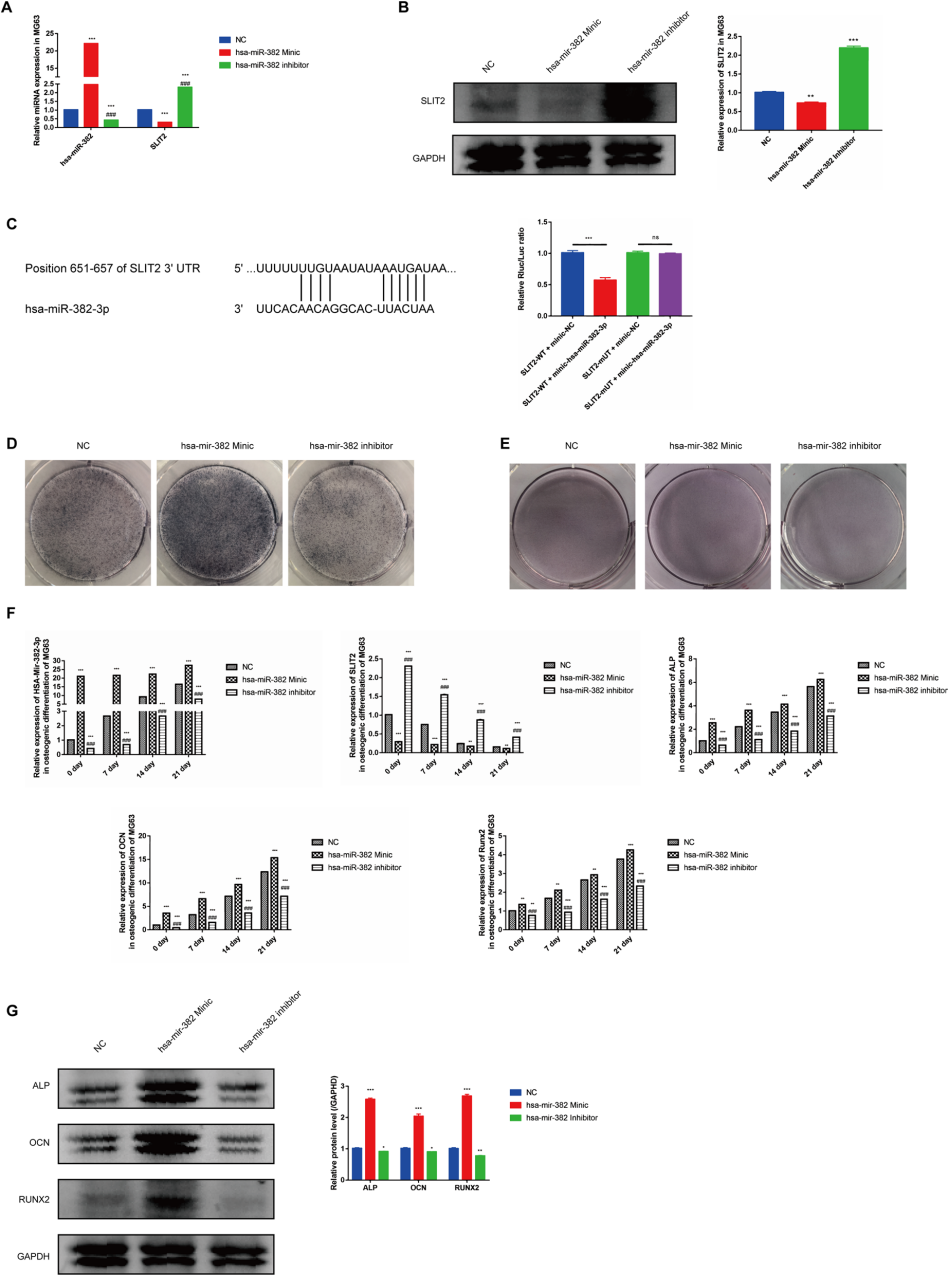
miR-382 promotes cell proliferation and osteogenesis in MG63 cell. **A**. qRT-PCR analysis of miR-382 level in MG63 cells after transfection with negative control, miR-382 mimic or miR-382 inhibitor. **B** Western blotting analysis of SLIT2 expression in MG63 cells after transfection with negative control, miR-382 mimic or miR-382 inhibitor. **C** Binding sites between miR-382 and SLIT2 gene predicted by TargetScan website (Left). The regulation of miR-382 on SLIT2 gene transcription is verified by dual-luciferase reporter gene assay (Right). **D** ALP staining, **E** alizarin red S staining, **F** qRT-PCR and **G** western blot analysis of miR-382, SLIT2 and osteogenesis-associated markers in MG63 cells after transfection with negative control, miR-382 mimic or miR-382 inhibitor. Statistical evaluation was performed using one-way ANOVA. Mean ± SEM. nsP > 0.05, *P < 0.05, **P < 0.01, ***P < 0.001. Statistical evaluation was performed using two-way ANOVA. Mean ± SEM. *represents *P* < 0.05 compared with NC group. *P < 0.05, ***P < 0.001; # represents *P* < 0.05 compared with HG group. ###*P* < 0.001.

Due to the increasing level of miR-382 in MG63 cell after internalization of hBMSC-sEV, we further investigated the role of miR-382 in osteogenesis. To identify which targeting protein was affected by the miR-382, we combined the results of PPI analysis and chose the SLIT2 as a candidate. Notably, when we over-regulated the expression of miR-382 through transfection of miR-382 mimic or applied miR-382 inhibitor to suppress its downstream signaling, the miR-382 level was increased after transfection, while inhibitor significantly down-regulated its express. Notably, the expression of SLIT2 was suppressed with up-regulation of miR-382 and was increased with treatment of miR-382 inhibitor, which indicated the targeting function of miR-382 on SLIT2 (Fig. 4A). Similar results were also observed in the protein level (Fig. 4B). According to the starBase online, we predicted the target potential between miR-382 and SLIT2 because of the complementary sequence. Next, through analysis by dual-luciferase reporter gene experiment, we concluded that over-expression of miR-382 inhibited SLIT2 expression. Mechanically, single-stranded miR-382 target mRNA sequences of SLIT2 by binding the 3′-untranslated regions (3′-UTR), resulting in the degradation or translation blockade of SLIT2 expression (Fig. 4C). Additionally, we found that up-regulation of miR-382 remarkably promoted osteogenesis by ALP staining and Alizarin red staining (Fig. 4D–E). According to the representatives of staining, the ability of osteogenic differentiation in MG63 cell was significantly enhanced. Similarly, osteogenic differentiation-associated genes and proteins were gradually increased over time (0, 7, 14 and 21 days) (Fig. 4F, G). Taken together, these results suggest that miR-382 could effectively promoted osteogenesis of osteoblast.

### SLIT2 mediated the pro-osteogenic function of miR-382-contained hBMSC-sEV

**Fig. 5:**
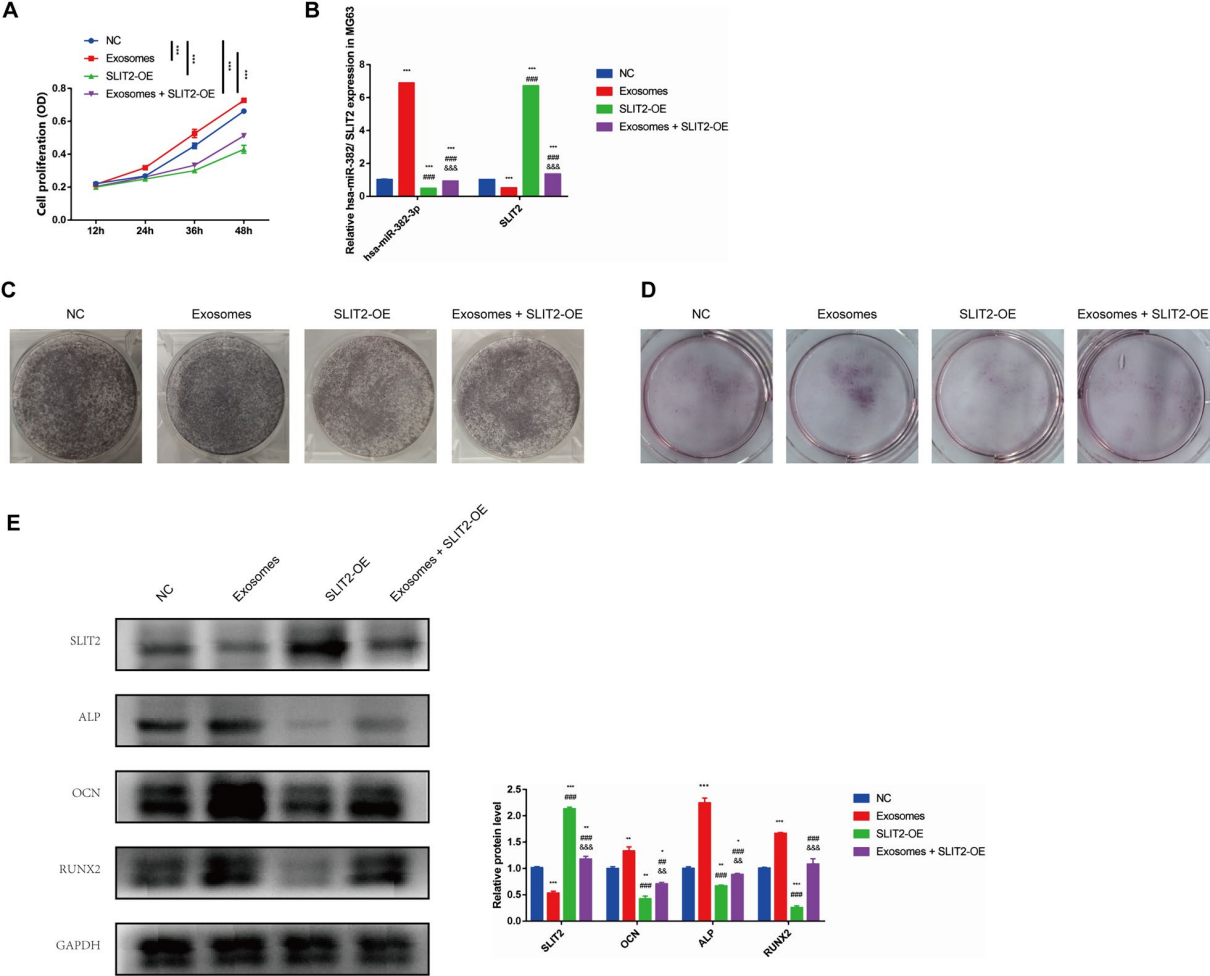
SLIT2 mediated the pro-osteogenic function of miR-382 in hBMSC-sEV. **A** CCK8 assays and **B** qRT-PCR of miR-382 and SLIT2 expression in MG63 cells treated with PBS or hBMSC-sEV after transfection of negative control or SLIT2 over-expression plasmid. **C** ALP staining, **D** alizarin red S staining and **E** western blot analysis of SLIT2 and osteogenesis-associated markers in MG63 cells treated with PBS or hBMSC-sEV following transfection of negative control or SLIT2 over-expression plasmid after osteogenic differentiation. Statistical evaluation was performed using two-way ANOVA. Mean ± SEM. *represents *P* < 0.05 compared with NC group. *P < 0.05, ***P < 0.001; # represents *P* < 0.05 compared with HG group. ###*P* < 0.001; &represents *P* < 0.05 compared with miR-182-5p group. &&*P* < 0.01, &&&*P* < 0.001

To further investigate the role in hBMSC-sEV-induced osteogenesis, we up-regulated the expression of SLIT2 in MG63 cell, combined with treatment of hBMSC-sEV. Then we detected the expression of miR-382 and SLIT2 after plasmid transfection and hBMSC-sEV treatment. According to the qRT-PCR and western blot, the SLIT2 level was significantly increased after over-regulation, which inhibited the expression of miR-382 following internalization (Fig. 5A, B). In addition, the cell proliferation assay presented that the promotive function on cell proliferation of hBMSC-sEV was abrogated by the up-regulation of SLIT2. Similarly, ALP staining, alizarin red S staining and western blot showed that the promoting role of hBMSC-sEV in osteogenesis was also inhibited through over-expression of SLIT2 (Fig. 5C–E). Taken together, SLIT2 mediated the inhibitive function on osteogenesis, which was the target of hBMSC-sEV-derived miR-382.

## Discussion

Osteoporosis (OP) is a systemic skeletal disease with clinical manifestations of increased susceptibility to bone fragility and fracture, pathologically characterized by low bone density and degeneration of bone tissue microstructure [35–37]. Moreover, osteoblast differentiation is of great importance in skeletal development and osteogenic progression. Dominantly, the imbalance between bone-resorbing and bone-forming osteoblasts may cause bone destructive diseases. Thus, grasping osteogenic differentiation not only provides insights into the bone development, but could also offer therapeutic strategies for OP.

Currently, hBMSC-derived sEV have been served as an effective strategies for a variety of disease with great characteristics, including low immunogenicity, easy storage and high biosafety, and have striking advantages over whole-cell therapy [38, 39]. It has been reported that EVs could be taken up via a variety of endocytic pathways, including macropinocytosis, CME, caveolin-mediated endocytosis and clathrin- and caveolin-independent endocytosis. In particular, sEV are commonly studied as a nanotherapeutic agent for stroke and wound-healing treatment. However, the curative effects and mechanisms of the action of hBMSC-sEV on osteogenesis are poorly understood, particularly in the osteoblast-mediated osteogenic differentiation. In our present study, we observed that hBMSC-sEV could promote osteogenic differentiation of osteoblast through miR-382 after internalization by targeting SLIT2.

The miRNAs are small, approximately 20 nucleotides long, noncoding, single-stranded RNA molecules and act as posttranscriptional regulators of gene expression [40]. It has been reported that the miR-382 family is crucial in multiple cancer types [41]. For instance, the invasion and proliferation ability of pancreatic cancer is significantly inhibited by miR-382 by targeting STAT1/PD-L1 [42]. In squamous cell carcinoma, miR-382 from cancer-associated fibroblast-derived exosomes promoted the metastasis and invasion [43]. Additionally, a previous study revealed that expression of the miR-382 family is significantly higher in glioma patients, compared with healthy person, and is crucial in cancer formation and progression [44]. Moreover, it has been reported that a reduction in mature miR-382 is associated with the proliferation and invasion of glioblastoma cells [45]. However, the miR-382-associated signaling network has not yet been explored in osteogenesis. In our study, we confirmed that hBMSC-sEV-derived miR-382 can remarkably increase its expression in osteoblast and effectively promote cell proliferation and osteogenesis.

Moreover, we assumed that SLIT2 may be a potential downstream of miR-382. SLIT2 plays the anti-inflammatory role in human placenta and decrease LPS-induced endothelial inflammation. SLIT2 is the most commonly studied protein among SLIT family and plays diverse roles in the migration of various types of cells, neural formation, angiogenesis and cancer progression [46–48]. Regarding bone metabolism, previous study reported that SLIT2 were significantly expressed in pre-osteoclasts and/or mature osteoclasts. Furthermore, it has been shown that SLIT2 inhibits osteoclast differentiation, mainly by reducing the migration and fusion of pre-osteoclasts, which are mediated by the suppression of Cdc42 activity [49]. However, the relationship between miR-382 and SLIT2 is still unknown.

The starBase online, an open-source platform for studying the interaction of miRNA and messenger RNA (mRNA), predicted the target potential between miR-382 and SLIT2 because of the complementary sequence. Therefore, we assumed that miR-382 may mediated the expression of SLIT2. Next, through analysis by dual-luciferase reporter gene experiment, we concluded that over-expression of miR-382 inhibited SLIT2 expression. Mechanically, single-stranded miR-382 target mRNA sequences of SLIT2 by binding the 3′-untranslated regions (3′-UTR), resulting in the degradation or translation blockade of SLIT2 expression. Specifically, the important role of microRNA in the formation of osteoblasts has been highlighted. miRNAs have been demonstrated to regulate OP progression by inducing or suppressing osteogenic differentiation, suggesting the relationship between dysregulation of osteogenic differentiation and OP.

Accumulating evidence has noted that patient with OP presented higher rates of bone resorption as well as impaired osteogenesis simultaneously. Notably, osteoblasts exert critical roles in bone formation through regulation on osteogenic differentiation-related proteins such as OCN, ALP and RUNX2. Our results demonstrated that up-regulation of miR-382 in hBMSC-sEV, which can be packaged and secreted into the microenvironment and internalized by osteoblast, can effectively target the expression of SLIT2 and mediated the expression of osteogenesis-associated proteins. Thus, we concluded that a miR-382-SLIT2 axis could exert a critical role in regulating the osteogenic differentiation potentials in OP patients.

In summary, using a series of in vitro experiments, we found poorly expressed miR-382 and high SLIT2 expression in OP patients. Specifically, over-expression of miR-382 or inhibition of miR-382 in hBMSCs could promote the osteogenic differentiation potentials of osteoblast through hBMSC-sEV-mediated cellular communication. Our study provided evidence that miR-382-contained hBMSC-sEV held great promise in osteogenic differentiation in osteoblast by targeting SLIT2, which can be served as molecular targets to develop effective therapy.
